# No-Reference Quality Assessment for 3D Synthesized Images Based on Visual-Entropy-Guided Multi-Layer Features Analysis

**DOI:** 10.3390/e23060770

**Published:** 2021-06-18

**Authors:** Chongchong Jin, Zongju Peng, Wenhui Zou, Fen Chen, Gangyi Jiang, Mei Yu

**Affiliations:** 1 Faculty of Information Science and Engineering, Ningbo University, Ningbo 315211, China; jinchongchong94@163.com (C.J.); zouwench@163.com (W.Z.); chenfen@cqut.edu.cn (F.C.); jianggangyi@126.com (G.J.); yumei@nbu.edu.cn (M.Y.); 2 School of Electrical and Electronic Engineering, Chongqing University of Technology, Chongqing 400054, China

**Keywords:** 3D synthesized images, image quality assessment (IQA), no-reference, visual-entropy-guided, multi-layer features analysis

## Abstract

Multiview video plus depth is one of the mainstream representations of 3D scenes in emerging free viewpoint video, which generates virtual 3D synthesized images through a depth-image-based-rendering (DIBR) technique. However, the inaccuracy of depth maps and imperfect DIBR techniques result in different geometric distortions that seriously deteriorate the users’ visual perception. An effective 3D synthesized image quality assessment (IQA) metric can simulate human visual perception and determine the application feasibility of the synthesized content. In this paper, a no-reference IQA metric based on visual-entropy-guided multi-layer features analysis for 3D synthesized images is proposed. According to the energy entropy, the geometric distortions are divided into two visual attention layers, namely, bottom-up layer and top-down layer. The feature of salient distortion is measured by regional proportion plus transition threshold on a bottom-up layer. In parallel, the key distribution regions of insignificant geometric distortion are extracted by a relative total variation model, and the features of these distortions are measured by the interaction of decentralized attention and concentrated attention on top-down layers. By integrating the features of both bottom-up and top-down layers, a more visually perceptive quality evaluation model is built. Experimental results show that the proposed method is superior to the state-of-the-art in assessing the quality of 3D synthesized images.

## 1. Introduction

With the advancement of video technologies, a free viewpoint video (FVV) system is gradually applied to various fields, such as distance education, medical service, and entertainment [1]. Compared with traditional 2D videos, users can interactively embody 3D scenes from arbitrary viewpoints in the FVV system. Unfortunately, limited by equipment and cost, capturing all views of FVV via camera is unrealistic and needs the existence of virtual synthesized viewpoints to enhance the scene switching continuity. Multiview video plus depth is one of the mainstream representations of 3D scenes, which generate virtual synthesized images through depth-image-based-rendering (DIBR) techniques [2]. At this stage, the inaccuracy of depth maps and imperfect DIBR techniques result in different geometric distortions which seriously deteriorate the users’ visual perception. In addition, it is time-consuming and impracticable to screen the quality of massive synthesized images by humans. Hence, designing an effective image quality assessment (IQA) metric [3] via human visual simulation to measure the image quality deterioration and further determine the application feasibility of 3D synthesized views is a significant research topic.

So far, extensive IQA methods were designed for the traditional distortions in 2D images, such as JPEG/JPEG2K compression [4,5], Gaussian white noise [6], Gaussian blur [7], blocking [8], and fast fading channel errors [9]. Generally, these distortions globally distribute in entire 2D images. In contrast, the 3D synthesized geometric distortions appear in local areas, and seriously destroy the structural semantic information of synthesized views. Due to the particularity of synthetic distortions, the existing IQA methods for 2D traditional distortions, like [10,11,12,13,14], cannot measure the 3D synthesized distortions effectively. With this concern, some researchers have proposed IQA metrics targeting 3D synthesized images. These methods are mainly divided into two categories, full-reference (FR) [15,16,17,18,19,20,21,22,23] and no-reference (NR) [24,25,26,27,28,29,30,31,32,33].

Bosc et al. explored the necessity of designing synthesized IQA metric, and evaluated the image quality via pixel deviation [15]. Conze et al. designed an SSIM-based view synthesis quality assessment (VSQA) metric, which mainly researched the synthesized view quality degradation caused by shift artifacts [16]. Battisti et al. statistically analyzed the shift artifacts in the Haar wavelet sub-bands, and proposed a 3D synthesized view image quality metric (3DSwIM) [17]. Ling and Le Callet proposed a sketch-token-based synthesized IQA (ST-SIQA) metric [18] and elastic metric based IQA (EM-IQA) metric [19]. Both ST-SIQA and EM-IQA analyzed shift artifacts by calculating contour similarity between the reference and synthesized images. Sandić-Stanković et al. designed two IQA metrics, i.e., morphological wavelet peak signal-to-noise ratio (MW-PSNR) [20] and morphological pyramid peak signal-to-noise ratio (MP-PSNR) [21], in order to evaluate the quality of synthesized geometric distortions in a transform domain. Tian et al. matched the horizontal displacement between the reference and synthesized images to devise a shift-compensation-based IQA (SC-IQA) metric [22]. Li et al. presented an FR quality metric for visual views by simultaneously measuring local instance degradation and global appearance (IDEA), in which local distortions were detected by discrete orthogonal moments and global sharpness was measured by super-pixel representation [23]. However, FR synthesized IQA metrics are not suitable for real application because the reference images of synthesized view are usually unavailable in FVV systems.

Gu et al. proposed an NR autoregression-plus thresholding (APT) metric based on a natural scene statistical (NSS) model [24]. Lately, Gu et al. considered local and global distortion, and presented a multi-scale NSS-based (MNSS) metric [25]. Jakhetiya et al. counted outliers by a three sigma rule-based robust outlyingness ratio (OUT) to evaluate the quality of synthesized images [26]. Recently, Jakhetiya et al. further proposed a kernel-ridge-regression-based predictor for synthesized IQA, which detected the complete distortion surface with geometric distortions and estimated corresponding quality scores [27]. The NSS-based methods above are time consuming and basically designed for severe geometric distortions. In addition, the metrics based on transform domain are also considered. Sandić-Stanković et al. proposed an NR IQA metric for synthesized videos which combined a high frequency component in a morphological wavelet domain with threshold (NR_MWT) [28]. Wang et al. also extracted features of geometric distortion, global sharpness, and image complexity in a wavelet transform domain to evaluate the quality of 3D synthesized images [29]. These transform-domain-based metrics eliminate uninterested information of synthesized image and save calculation time but are still sensitive to limited geometric distortion types. Based on this, Zhou et al. analyzed synthesized images using Difference-of-Gaussian-based edge statistics and texture naturalness (SET) to measure different types of geometric distortions [30]. Tian et al. proposed an NR IQA of synthesized views (NIQSV), which measured the blurry and crumbling distortions by opening and closing operations [31]. Subsequently, Tian et al. further analyzed the hole and stretching distortions, and advanced the NIQSV to NIQSV+ [32]. Likewise, Yue et al. classified the distortions, and combined local and global features to measure 3D synthesized images (CLGM) [33]. These distortion-classification-based metrics targeted measure multiple distortion types and are more comprehensive. The pity is that the synthesized image degradation caused by weak geometric distortions has not received enough attention. Furthermore, few deep-learning-based metrics were exploratively used to evaluate the quality of 3D synthesized images. Ling et al. proposed a generative adversarial networks-based NR metric (GANs-NRM) for synthesized images, which expanded the distortion sample through the GANs, then used a ‘bag of distortion word’ codebook to classify the distortion, and finally used the support vector machine to regress the quality score [34]. However, it only uses the network to expand the training samples and does not achieve end-to-end score learning. Wang et al. built a new synthesized database including 504 pictures to expand the ground-truth of training and utilized the local saliency to weight the predicted scores [35]. Unfortunately, the database samples proposed by this method are still limited. Thus, how to evaluate the synthesized images using an end-to-end deep learning model though the small database still remains an open problem.

In summary, the existing IQA metrics for 3D synthesized images still have some limitations. (1) The reference images are not accessible in the FVV system. (2) Most of the existing IQA metrics search geometric distortions though the entire image, which have difficulty measuring local-distributed distortions in synthesized images. (3) Although the performance of the distortion-classification-based IQA metrics is competitive, they have room for further improvement in terms of weak geometric distortion measurement.

In this paper, a novel NR IQA metric based on visual-entropy-guided multi-layer features analysis (MLFA) is proposed. Extensive experiments exhibit that MLFA has a better performance than the prevailing IQA metrics and strong robustness on different databases. The main contributions of MLFA are as follows:

(1) The metric elaborately classifies geometric distortions into bottom-up and top-down layers via visual entropy, and integrates multi-layer features to regress quality score.
(2) In the bottom-up layer, the strong geometric distortion is measured by calculating area proportion plus transition threshold.
(3) In the top-down layer, key regions of weak geometric distortions are extracted by the relative total variation model, and the features are measured by the interaction of decentralized attention (entropy, secondary Gaussian blur similarity, and horizontal pixels correlation) and concentrated attention (Gaussian mixture models).

The rest of this paper is organized as follows. The motivation of our method is detailed in Section 2. Section 3 describes the visual-entropy-guided MLFA method for synthesized images. Section 4 presents the experimental results. Finally, conclusions are drawn in Section 5.

## 2. Motivation

**Figure 1:**
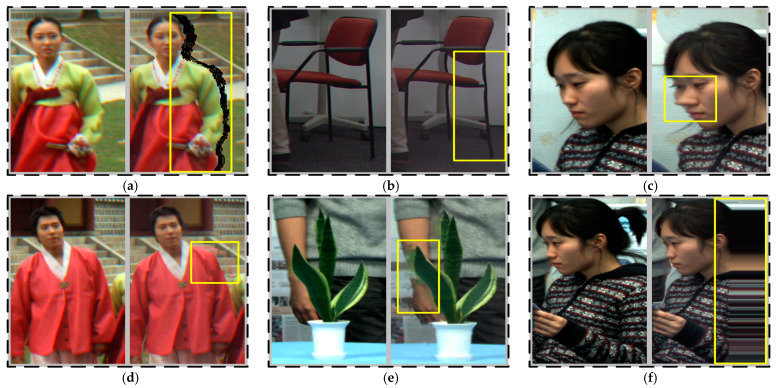
3D synthesized geometric distortions, (**a**) hole; (**b**) curving; (**c**) object shifting; (**d**) ghosting; (**e**) blurry; (**f**) stretching.

Figure 1 shows the visual comparison pair of geometric distortions, the left and right are the local areas of original and distorted images, respectively, and all subfigures are originated from the IRCCyN_IVC_DIBR_images database [36]. Different from traditional 2D distortions, 3D synthesized geometric distortions are mainly caused by inaccurate depth map and DIBR techniques.

Figure 1a shows the hole distortion. Occlusion and exposure are the main reasons for the hole generation. If one object is occluded in the real view and exposed in the virtual view, the corresponding region in virtual view cannot be warped from the real view. Consequently, a hole is generated. Most of the hole phenomenon occurs in the depth abrupt areas.

To tackle the hole problem, many scholars preprocessed the depth video. For instance, Fehn et al. [37] used a low-pass filter to smooth the depth information. By this method, the hole problem can be alleviated in synthesized images, but inaccurate depth information also brings the geometric distortions, curving, and object shifting, which are respectively visible in the chair and face of Figure 1b,c. One can see that the distortions are particularly perceptible in background and foreground transitions.

In addition, the filling algorithms for hole regions also bring distortions. Figure 1d shows the ghosting phenomenon based on patch-based synthesis methods [38,39]. This distortion generates when the pixels from the optimal matching patch do not fit the actual scene. Figure 1e shows a rendered result when the hole area is filled using the methods in [40,41]. As we can observe, the in-painting method cannot effectively fill holes in complex texture areas, which result in blurry distortion at the boundaries of the potted plant and the man’s arm. Additionally, the stretching distortion mainly occurs on the left/right side of image and is produced by a particular in-painting method [42], which fills holes with existing horizontal adjacent pixels, as shown in Figure 1f.

According to the observations of above synthesized distortions, we find that people cannot distinguish the specific distortion types without professional training, and can only roughly evaluate the degree of image quality degeneration. Therefore, the mess types of synthesized distortions are regulated for unified measurement. The distortions, caused by inaccurate depth information, i.e., curving and object shifting, are classified to ‘deforming’. The distortions that are manifested as the pixel overflow and caused by the inaccurate filling algorithm, i.e., ghosting and blurry, are collectively called ‘blurry’. Simultaneously, we find that the geometric distortion often occurs in the local areas of synthesized images, especially on the left/right side of images and the boundary areas of objects.

Biologically, visual stimuli enter the primary visual cortex for the short term and progress along two parallel hierarchical streams, i.e., the brain neurons are divided into two major regions to control the attention mechanism. The ‘dorsal stream’ mainly processes visual information in the posterior parietal cortex and is concerned with directing attention. The ‘ventral stream’ processes stimuli in the inferotemporal cortex, focusing on recognition capability [43]. The dorsal and ventral streams must interact to achieve good scene understanding. However, the fusion of two streams to process information is simple for the human brain, but challenging for the computer. Otherwise, implementing two streams at the same time has an obstacle that only small parts of visual stimuli are stored as short-term memory [44]. Hence, processing a large amount of sensory information in one step is unrealistic.

We focus our research by combining biological theories, and the distinction of energy entropy included in different stimuli (i.e., distortions) is huge, which may cause different distortions to be processed in different visual cortexes. This presumption is indeed verified by some studies—that there exists an approximately linear relationship between energy entropy and a visual attention mechanism [45,46]. Thus, a two-component framework for visual attention mechanism stimulated by stimuli energy entropy (short for visual entropy) was proposed to simulate the physiological structure of the human brain processing visual information [47]. The framework suggests a human selective attention scene though bottom-up and top-down mechanisms. The bottom-up mechanism means that a stimulus with high energy is sufficiently salient and can pop out of a visual scene, which will take 20–50 ms reaction time of human attention. On the contrary, for the top-down mechanism, like a task in which people need to move their eyes to find low energy scenes, such volitional attention will take 200 ms or more reaction time. Inspired by this theory, hole distortion can pop out in an image due to its obviousness, which tends to be a bottom-up mechanism. Other distortions are interfered by complex textures and require careful observation, which takes a longer reaction time and tends to be a top-down mechanism. In addition, the visual attention mechanism is affected by inhibition of return (the current attention will not be attended again), so both bottom-up and top-down mechanisms can operate in parallel.

Particularly, the performance of top-down attention is controlled by complex brain regions, such as the frontal lobes. Hence, it is difficult to express visual perception by integration of the various scene features. Treisman and Gelade proposed a feature integration theory [48], which came up with two visual attention mechanisms, decentralized attention and concentrated attention. The former is a decentralized search for different features of the scene (e.g., color, shape). The latter is mainly a concentrated search for the scene where various features are mixed. The decentralized attention is a single-dimensional feature extraction, which has strong pertinence and information dependence. By contrast, the concentrated attention is a multi-dimensional extraction of mixed features, which has strong robustness to information update. Therefore, we consider extracting the distortions of the top-down layer via feature integration theory (i.e., decentralized and concentrated attention) to achieve the maximum utilization of features.

Based on all distortion observations, biology and psychology theory, we divide the 3D synthesized geometric distortions into two visual-entropy-guided attention layers. Specifically, the hole distortion is divided into a bottom-up layer because of its eye-catching energy, and insignificant geometric distortions (i.e., deforming, blurry, and stretching) are assigned to a top-down layer. Further focusing on a top-down layer, the key distributed areas (i.e., left/right side of images and the boundary areas of objects) of weak geometric distortion are highlighted, and the decentralized and concentrated attention are combined to measure top-down features based on key areas. By integrating the features of bottom-up and top-down layers, a novel NR IQA metric for 3D synthesized images is built. Extensive experimental results demonstrate the effectiveness and robustness of the proposed method (MLFA).

## 3. The Proposed Visual-Entropy-Guided MLFA Method

**Figure 2:**
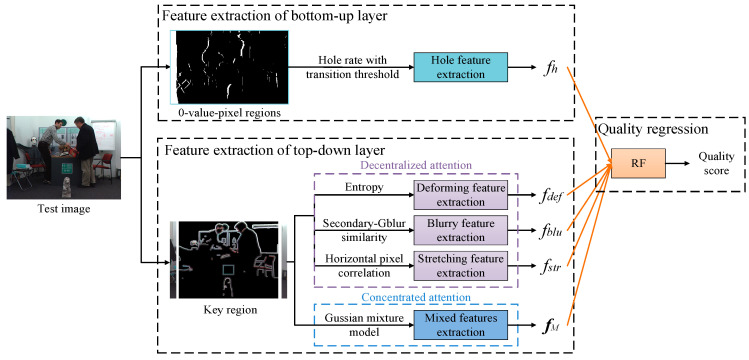
Block diagram of the visual-entropy-guided MLFA method.

Figure 2 shows the block diagram of the proposed visual-entropy-guided MLFA method, which contains three parts, feature extraction of bottom-up and top-down layers, and quality regression of random forest (RF). The details of each part will be introduced in Section 3.1, Section 3.2 and Section 3.3.

### 3.1. Feature Extraction of the Bottom-Up Layer

**Figure 3:**
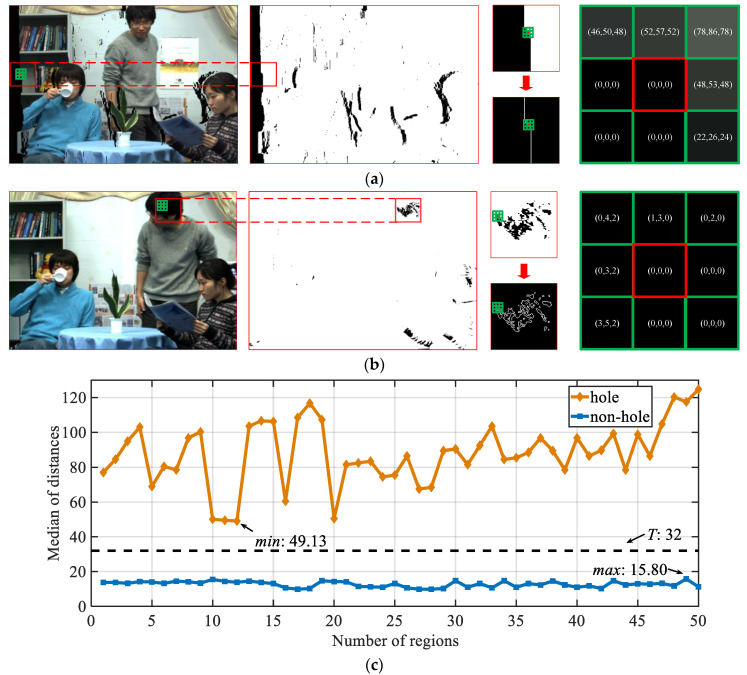
Distinguish the hole and non-hole regions. (**a**) 3D synthesized image with 0-value-pixel regions (hole regions); (**b**) original image with 0-pixel regions (non-hole regions); (**c**) median of distances about the hole regions (orange line) and non-hole regions (blue line).

Figure 3 shows two kinds of black areas origination in the IRCCyN_IVC_DIBR_images database: natural black object and hole distortion, in which the natural black object does not affect the quality attenuation of the image. Therefore, we need to eliminate the interference caused by natural black objects (i.e., non-hole) when extracting the hole features. Specifically, the regions with a pixel value of 0 are calculated as the candidate areas, as shown in the second subfigures of Figure 3a,b.

Subsequently, we find that, compared with non-hole 0-pixel regions, the boundary pixels of hole regions are more abrupt, such as the rightmost subfigures of Figure 3a,b shown. Thus, a statistical method of boundary-pixel transition is introduced. The 0-value-pixel boundaries are obtained by Sobel algorithm (visualized in the third column subfigures of Figure 3a,b, and the Euclidean distance *d* between the current boundary pixel and the predicted boundary pixel are calculated:

(1) $$d\left(i,j\right)=|b\left(i,j\right)-\tilde{b}\left(i,j\right)|$$

where *b*(*i*,*j*) represents 0-value boundary pixels at (*i*,*j*). $\tilde{b}\left(i,j\right)$ means the pixel value predicted by the transition statistical method at (*i*,*j*):

(2) $$\tilde{b}\left(i,j\right)=\frac{1}{n\times n}\underset{q\left(i,j\right)\in \Omega 1}{\sum }q\left(i,j\right)$$

where *q*(*i*,*j*) belongs to **Ω1**, which are adjacent pixels surrounding (*i*,*j*) in the *n* × *n* patch.

After that, the same numbers of hole and non-hole 0-pixel regions are respectively selected to get their median of distances as shown in Figure 3c. Based on the size of database [36], the number of 0-pixel regions are set to 50, and the median performance of 1000 calculations is considered as the model to exclude outlier distances. Then, a transition threshold is defined as *T* = *Average* (*min* {hole}, *max* {non-hole}) to distinguish between hole and non-hole regions. Here, *T* is rounded to 32.

To the end, the hole rate is calculated as the feature of the bottom-up layer:

(3) $$f_{h}=\{\begin{array}{cc} \frac{Num\left(R_{h}\right)}{W\times H} & Median\left\{d\left(i,j\right)\right\}>T\\ 0 & otherwise \end{array}$$

where *Num*(·) indicates the pixel number. **R***_h_* represents hole regions. *W* and *H* denote the width and height of the test image. 

### 3.2. Feature Extraction of a Top-Down Layer

**Figure 4:**
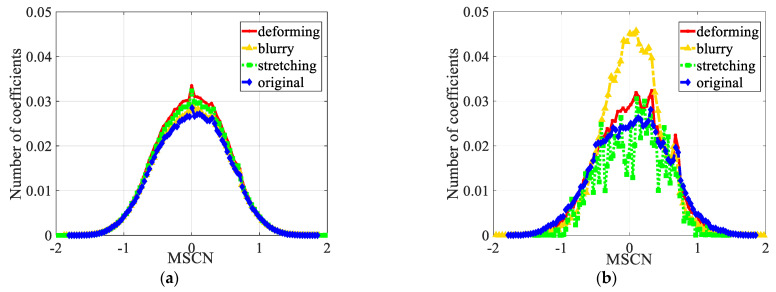
The histograms of the MSCN coefficients of the original image and its corresponding top-down distorted versions. (**a**) MSCN in unprocessed images; (**b**) MSCN in key region **R***_k_*.

As mentioned by the APT metric [24], the performance of using the NSS model directly on unprocessed synthesized images is poor, and the histograms of different geometric distortions are quite close to each other as shown in Figure 4a. Thus, to avoid the global ‘good quality’ information affecting local distorted information, popping out the local distorted regions is indispensable. Due to the distortions that usually occur on the left/right side of the image and boundaries of objects, we consider extracting these two parts as the key region. Fundamentally, the test image is divided into the side region (**R***_s_*) and middle region (**R***_m_*) according to the image width (*W*):

(4) $$\{\begin{array}{l} R_{m}\left(i,j\right)=\left\{Y\left(i,j\right)|T_{l}\cdot W\leq i\leq \left(1-T_{r}\right)\cdot W,\;0\leq j\leq H\right\}\\ R_{s}\left(i,j\right)=Y\left(i,j\right)-R_{m}\left(i,j\right) \end{array}$$

where **Y**(*i*,*j*) is the pixel value at (*i*,*j*) in the test image. *T_l_* and *T_r_* are width proportion thresholds, which determine the left and right sides of **R***_m_* in the image.

Further to **R***_m_*, inspired by the fact that the image semantic contains structure and texture information [49], we combine image structure extraction with morphological operations to extract the boundaries of objects. Specifically, a relative total variation model is used to extract the structure image **S**:

(5) $$\arg\underset{s\left(i,j\right)}{\min}\underset{i,j}{\sum }{\left(S\left(i,j\right)-R_{m}\left(i,j\right)\right)}^{2}+\lambda \cdot \left(\frac{W_{s,x}\left(i,j\right)}{W_{f,x}\left(i,j\right)+\varepsilon }+\frac{W_{s,y}\left(i,j\right)}{W_{f,y}\left(i,j\right)+\varepsilon }\right)$$

where the first term aims to make **S**(*i*,*j*) and **R***_m_*(*i*,*j*) similar. *λ* is a weight which determines the blur degree of the structure image. *ε* is a small constant to avoid the situation of division-by-zero. $W_{s,x}\left(i,j\right)$ and $W_{s,y}\left(i,j\right)$ are the values measured by sliding window in *x* and *y* directions:

(6) $$\{\begin{array}{c} W_{s,x}\left(i,j\right)=\sum_{k\left(i,j\right)\in \Omega 2}g_{\left(i,j\right),k\left(i,j\right)}\cdot \left|{\left(\partial_{x}S\right)}_{k\left(i,j\right)}\right|\\ W_{s,y}\left(i,j\right)=\sum_{k\left(i,j\right)\in \Omega 2}g_{\left(i,j\right),k\left(i,j\right)}\cdot \left|{\left(\partial_{y}S\right)}_{k\left(i,j\right)}\right| \end{array}$$

where *k*(*i*,*j*) belongs to **Ω2**, the 3 × 3 neighboring pixels centered at (*i*,*j*). ∂x and ∂y are partial derivatives. *g*_(*i*,*j*),*k*(*i*,*j*)_ is a weighting function, which is proportional to the exponent:

(7) $$g_{\left(i,j\right),k\left(i,j\right)}\propto \exp(-\frac{{\left(x_{\left(i,j\right)}-x_{k\left(i,j\right)}\right)}^{2}+{\left(y_{\left(i,j\right)}-y_{k\left(i,j\right)}\right)}^{2}}{2\sigma_{s}{}^{2}})$$

where *σ_s_* dominates the scale of the window and controls the scale of the texture element.

Similarly, $W_{f,x}\left(i,j\right)$ and $W_{f,y}\left(i,j\right)$ are measured by a fixed window in Equation (5). They are defined as:

(8) $$\{\begin{array}{c} W_{f,x}\left(i,j\right)=\left|\sum_{k\left(i,j\right)\in \Omega 2}g_{\left(i,j\right),k\left(i,j\right)}\cdot {\left(\partial_{x}S\right)}_{k\left(i,j\right)}\right|\\ W_{f,y}\left(i,j\right)=\left|\sum_{k\left(i,j\right)\in \Omega 2}g_{\left(i,j\right),k\left(i,j\right)}\cdot {\left(\partial_{y}S\right)}_{k\left(i,j\right)}\right| \end{array}$$

Different from the formula in Equation (6), the value obtained by a fixed window does not include the modulus. Thus, the sum of *∂*_(·)_*S* directly decides the gradient consistency.

In short, the structure and texture information of **S** depends on two parameters: λ and *σ_s_*. When λ and *σ_s_* are small, **S** contains complex texture information. Otherwise, details of **S** are lost too much to capture object boundaries reasonably. Here, *λ* and *σ_s_* are experimentally set as 0.02 and 4.

**Figure 5:**
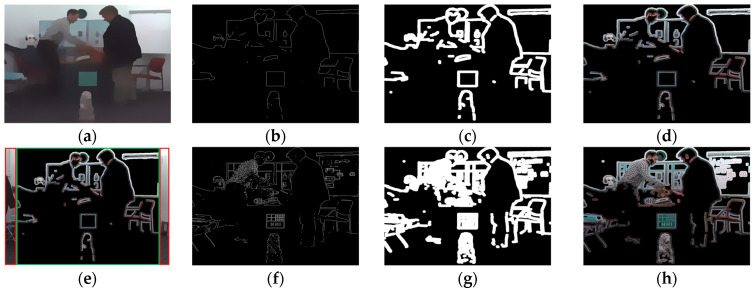
Illustration and visual comparison of key region extraction. (**a**) structure image **S**; (**b**) structure edge image **S***_e_*; (**c**) structure mask image **S***_m_*; (**d**) structure distortion image **S***_d_*; (**e**) key region **R***_k_*; (**f**) original edge image **O***_e_*; (**g**) original mask image **O***_m_*; (**h**) original distortion image **O***_d_*.

Figure 5 presents the visualized results of the relative total variation model with morphological processing. Specifically, Figure 5a shows the acquired structure image **S**. The structure edge image **S***_e_* and structure mask image **S***_m_* are obtained by the Sobel algorithm and dilation operation, as shown in Figure 5b,c. Figure 5d shows the structure distortion image **S***_d_*, which is obtained by **S** × **S***_m_*. Finally, the key region **R***_k_* is stitched by **R***_s_* (red boxes in Figure 5e) and **S***_d_* (green box in Figure 5e). In addition, the original edge image **O***_e_*, the original mask image **O***_m_*, and the original distortion image **O***_d_* are calculated for comparison as shown in Figure 5f–h. It can be found that **O***_d_* is more complicated and chaotic than **S***_d_*, which proves that the extracted **R***_k_* can effectively highlight object boundaries’ regions with geometric distortion and is consistent with the subjective perception of real synthesized distortion regions.

After the **R***_k_* is extracted, the feature integration theory (i.e., decentralized and concentrated attentions) is applied to measure the geometric distortions on the top-down layer. On the one hand, the features of geometric distortions on top-down layer are independently extracted by decentralized attention.

For the deforming distortion, it can be observed from Figure 1b,c that the regular pixel arrangement turns to a disorderly distribution after deforming. As a universal cognition, image entropy is a quantity that expresses the degree of disorder of the pixels state. Therefore, we use image entropy to extract the feature of deformation:

(9) $$f_{def}=-\underset{a}{\sum }\underset{b}{\sum }P_{a,b}\mathrm{lg}P_{a,b}$$

where *a* is the gray value of the pixel, and *b* is the average gray value in the 3 × 3 neighborhood. *p* = *f*(*a*,*b*)/*Num*(**R***_k_*) expresses the frequency that the gray feature group ***f***(*a*, *b*) in **R***_k_*.

For the blurry distortion, since its distortion appearance is similar to Gaussian blur (Gblur), a secondary Gblur plus structural similarity (SSIM) [11] is calculated as the feature:

(10) $$f_{blu}=S\left(R_{k}\left(i,j\right),R_{k}^{\prime \prime }\left(i,j\right)\right)=\frac{2R_{k}\left(i,j\right)\cdot R_{k}^{\prime \prime }\left(i,j\right)+\varepsilon }{R_{k}^{2}\left(i,j\right)+R_{k}^{\prime \prime }{}^{2}\left(i,j\right)+\varepsilon }$$

where $R_{k}^{\prime \prime }\left(i,j\right)=R_{k}\left(i,j\right)\cdot w\left(i,j\right)$ is the secondary Gblur image; among this, the value of Gaussian weight $w\left(i,j\right)=\frac{1}{2\pi \sigma_{b}{}^{2}}\exp(-\frac{i^{2}+j^{2}}{2\sigma_{b}{}^{2}})$, and *σ_b_* = 1.5.

For the stretching distortion, the horizontal pixel correlation is analyzed. Specifically, we detect the value equality of current pixel and its horizontal neighboring pixels. If the pixel satisfies the relevance condition, the numbers of pixels are counted:

(11) $$f_{str}=\{\begin{array}{cc} \frac{Num\left(x\left(i,j\right)\right)}{R_{k}\left(i,j\right)} & \sum_{l=1}^{2}\|x\left(i+l,j\right)-x{\left(i,j\right)\|}_{1}=0\\ 0 & otherwise \end{array}$$

where *x*(*i*,*j*) denotes the pixel value at pixel coordinates (*i*,*j*). 

On the other hand, the mixed multi-dimensional features are concentratively extracted by the Gaussian mixture model. The image is normalized:

(12) $$R_{k}^{\prime }\left(i,j\right)=\frac{R_{k}\left(i,j\right)-\mu_{k}\left(i,j\right)}{\sigma_{k}\left(i,j\right)+\varepsilon }$$

where *μ_k_*(*i,j*) and *σ_k_*(*i,j*) are the mean and contrast value of **R***_k_*(*i*,*j*), which are calculated by a Gaussian kernel with a size of 3 × 3. $R_{k}^{\prime }\left(i,j\right)$ represents the mean subtracted contrast normalized (MSCN) coefficient.

Figure 4b plots a histogram of MSCN coefficients for an original image and different top-down geometric distorted versions to visualize how the MSCN coefficient distributions change as a function of geometric distortions. Compared with Figure 4a, the MSCN coefficients can explicitly distinguish different top-down distortions within a certain range in key region **R***_k_*, which further verify the effectiveness of the above-mentioned key region extraction strategy.

In addition, the MSCN coefficients of adjacent pixels also have similar statistical characteristics. The MSCN coefficients of the present pixel and its four adjacent pixels (horizontal, vertical, main-diagonal, and secondary-diagonal) are calculated. Then, the Gaussian mixture model, which consists of generalized Gaussian distribution (GGD) and asymmetric GGD (AGGD), is used to extract mixed multi-dimensional features [50]. The mixed feature *f_M_* is a set with **f***_GGD_* and **f***_AGGD_*:

(13) $$f_{GGD\left(x;\alpha ,\sigma^{2}\right)}=\frac{\alpha \sqrt{\Gamma \left(3/\alpha \right)}}{2\sigma \sqrt{\Gamma^{3}\left(1/\alpha \right)}}\exp(-\left(\frac{\left|x\right|}{\sigma }\cdot \sqrt{\frac{\Gamma \left(3/\alpha \right)}{\Gamma \left(1/\alpha \right)}}{)}^{\alpha }\right)$$

(14) $$f_{AGGD\left(x;\beta ,\sigma_{l},\sigma_{r}\right)}=\{\begin{array}{cc} \frac{\beta \sqrt{\Gamma \left(3/\beta \right)}}{\left(\sigma_{l}+\sigma_{r}\right)\sqrt{\Gamma^{3}\left(1/\beta \right)}}\exp(-\left(\frac{-x}{\sigma_{l}}\sqrt{\frac{\Gamma \left(3/\beta \right)}{\Gamma \left(1/\beta \right)}}{)}^{\beta }\right) & x<0\\ \frac{\beta \sqrt{\Gamma \left(3/\beta \right)}}{\left(\sigma_{l}+\sigma_{r}\right)\sqrt{\Gamma^{3}\left(1/\beta \right)}}\exp(-\left(\frac{-x}{\sigma_{r}}\sqrt{\frac{\Gamma \left(3/\beta \right)}{\Gamma \left(1/\beta \right)}}{)}^{\beta }\right) & x\geq 0 \end{array}$$

where $\Gamma \left(x\right)=\int_{0}^{\infty }t^{x-1}e^{-t}dt,x>0$, *α* and *σ*^2^ are the parameters of *GGD*, which reflect the shape and variance features of the current pixel distribution. *β*, *σ_l_*, *σ_r_*, and *η* are four parameters that affect *AGGD*. The *AGGD* gets the best performance when $\eta =\left(\sigma_{r}-\sigma_{l}\right)\frac{\Gamma \left(2/\beta \right)}{\sqrt{\Gamma \left(3/\beta \right)}\sqrt{\Gamma \left(1/\beta \right)}}$.

In addition, owing to the human perception for scenes being multi-scale [25], we build the feature extraction model on original and down-sampled images. Therefore, the Gaussian mixture model generates 36-dimensional features, which includes *α* and *σ*^2^ in *GGD* and *β*, *σ_l_*, *σ_r_*, and *η* in *AGGD* with four adjacent directions and two image scales, i.e., *f_M_ =* [4**f***_GGD_*, 32**f***_AGGD_*].

### 3.3. Quality Regression

In this part, we use the regression function *H_m_*(·) to map the extracted features to objective scores *Q*, which are expressed as:

(15) $$Q=H_{m}\left(f_{total}\right)$$

where *H_m_*(·) is obtained by machine learning, and **f***_total_* = [*f_h_*, *f_def_*, *f_blu_*, *f_str_*, *f_M_*] are the total feature vectors.

RF shows favorable accuracy and has few over-fitting problems in regression operator. Therefore, we use the RF to learn the function *H_m_*(·) and achieve the predication of objective quality scores. In specific experiments, the 3D synthesized images in databases are divided into two non-overlapping parts randomly, 80% are used for training and the rest 20% are used for testing. The process of ‘training-testing’ is repeated for 1000 times, and the median performance is selected as the final model to eliminate performance bias.

## 4. Experimental Results and Analysis

This section mainly evaluates the performance of the visual-entropy-guided MLFA method. Firstly, we introduce the databases and performance evaluation criteria used in experiments. Secondly, the parameters are determined for achieving the best performance. Then, the performance of the visual-entropy-guided MLFA method is compared with other state-of-the-art metrics. Finally, generalization ability, impact of training percentages, multi-layer strategy, key region extraction strategy, and feature ablation experiments are implemented to prove the effectiveness of the visual-entropy-guided MLFA method.

### 4.1. Databases and Evaluation Criteria

Two databases, IRCCyN_IVC_DIBR_images database [36] and IETR DIBR image database [51], are used for the experiment in this study. The IRCCyN_IVC_DIBR_images database contains 96 images (84 3D synthesized images, 12 original images) and subjective quality scores. The database has three sequences, each sequence has four virtual views synthesized by a neighboring viewpoint using seven DIBR algorithms [37,38,39,40,41,42]. The IETR DIBR image database contains 150 images (140 3D synthesized images, 10 original images) and subjective quality scores. The database uses seven updated DIBR algorithms [52,53,54,55,56,57,58] to synthesize visual views and excludes some old-fashioned distortions (e.g., hole). 

Pearson Linear Correlation Coefficient (PLCC), Spearman Rank Correlation Coefficient (SRCC), and Root Mean Square Error (RMSE) are used to evaluate the difference between objective scores from metrics and subjective scores. The higher value of PLCC and SRCC, and the lower value of RMSE, mean that objective scores predicted by metrics are more similar to the subjective scores.

### 4.2. Parameters Determination

Three parameters, *n*, *T_l_*, and *T_r_* in the MLFA method, are determined in the IRCCyN_IVC_DIBR_images database.

Table 1 lists *n* and the corresponding discrimination performance of hole and non-hole regions. Specifically, we set *n* = {3, 5, 7, 9, 11} and select 50 hole and non-hole regions respectively to calculate each median of distances. Next, the standard deviation is used to compare the stability of 50 regions. The smaller value of standard deviation means more stable performance. Then, *T* and computational time are calculated in different values of *n*. The experimental results show that, with the increase of *n*, the standard deviation of the hole increases slightly, but the standard deviation of non-hole increases dramatically. This unstable trend decreases the distinction between hole and non-hole areas, and eventually *T* cannot be obtained. In addition, the method also becomes time-consuming with the increase of *n*. In short, the experimental data verify that expanding the value of rectangle adjacent pixels will destruct the autocorrelation of transition statistics between hole and non-hole regions and increase computational complexity. Hence, in the MLFA method, we assign *n* as equal to 3.

**Figure 6:**
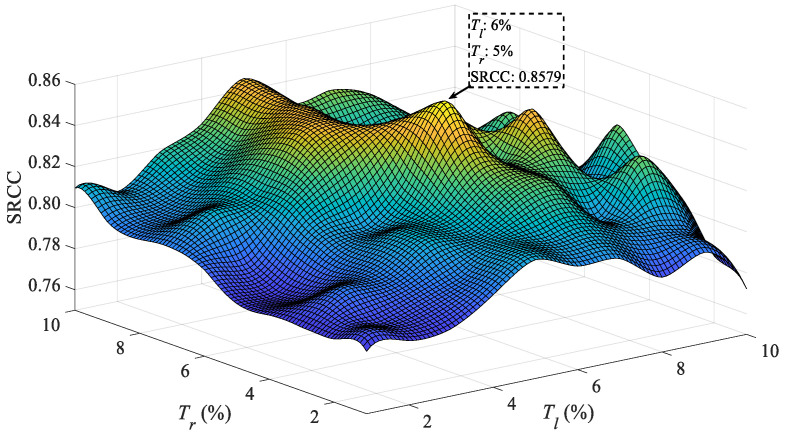
SRCC with different *T_l_* and *T_r_*.

Figure 6 shows the impact of different width thresholds *T_l_* and *T_r_* on SRCC performance. The optimal thresholds are determined by comparing the SRCC values when the ranges of *T_l_* and *T_r_* are 0 to 10. Form 3D surface of SRCC performance, the *T_l_* and *T_r_*, with largest SRCC (SRCC = 0.8579), are 6% and 5%, respectively. Hence, we set *T_l_* as 6%, and *T_r_* as 5% in the proposed MLFA method.

### 4.3. Performance Comparison

Table 2 illustrates the PLCC, SRCC, and RMSE performance comparison of MLFA with state-of-the-art metrics on the IRCCyN_IVC_DIBR_images database in which the best performance is highlighted with bold font. Specifically, the PSNR and SSIM [11] are traditional IQA metrics. The VSQA [16], 3DSwIM [17], ST-SIAQ [18], EM-IQA [19], MW-PSNR [20], MP-PSNR [21], SC-IQA [22], and IDEA [23] are FR IQA metrics for 3D synthesized images. The APT [24], MNSS [25], OUT [26], NR-MWT [28], SET [30], NIQSV [31], NIQSV+ [32], CLGM [33], GANs-NRM [34], and Wang [35] are NR metrics designed for 3D synthesized images. In the experimental results, we can obtain three potential conclusions:

(1) The traditional metrics, like PSNR and SSIM, are not effective for 3D synthesized images. The performance of PSNR and SSIM is poor because they have not been conceived for dealing with the local specificity of geometric distortions (e.g., the PLCC is less than 0.5).
(2) The performance of metrics designed for 3D synthesized images is better than traditional metrics, but not sufficient. The metrics, VSQA, 3DSwIM, ST-SIAQ, EM-IQA, and NIQSV, are mainly designed for the object shifting and blurry distortions (parts of the top-down layer). The metrics, MW-PSNR, MP-PSNR, APT, MNSS, and OUT, are mainly sensitive to hole distortion. The above-mentioned metrics ignore the diversity of geometric distortions. Among them, the MNSS metric shows the best performance, and PLCC, SRCC, and RMSE are 0.7700, 0.7850, and 0.4120. A few metrics consider multiple distortions, such as SC-IQA, IDEA, NR-MWT, SET, NIQSV+, and CLGM. However, the weak geometric distortions are inadequately and ambiguously classified, and merely measured via decentralized attention. In addition, only a few metrics (e.g., IDEA) emphasize the utilization of local distortion distribution characteristics. These limitations lead these metrics to fail to effectively estimate weak distortions. Even for SET, the best among these metrics, the corresponding PLCC, SRCC, and RMSE are 0.8586, 0.8109, and 0.3015, and can be further improved. The performance of deep-learning-based metrics, such as GANs-NRM and Wang, is also unsatisfactory due to the limitation of insufficient training samples.
(3) The proposed method MLFA is superior to the state-of-the-art metrics, and PLCC, SRCC, and RMSE are 0.8757, 0.8579, and 0.4106. It affirms the effectiveness of MLFA method for 3D synthesized images.

**Figure 7:**
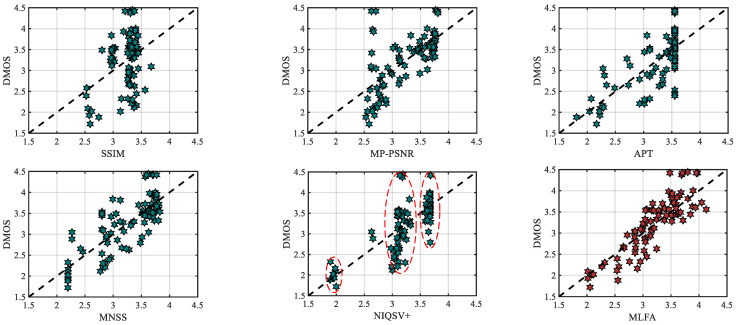
Scatter plots of subjective DMOS and objective scores on the IRCCyN_IVC_DIBR_images database.

Figure 7 shows the scatter plots of subjective DMOS and objective scores in SSIM, MP-PSNR, APT, MNSS, NIQSV+, and MLFA on the IRCCyN_IVC_DIBR_images database. The points of the MLFA method aggregate on the fitting line. By contrast, the scattered plots of the comparative metrics present vertical point distribution, i.e., objective scores of the vertical distributed points are similar, while the subjective scores are different. By validating the corresponding image of each point, we found that the comparative metrics can roughly distinguish specific distortions but fail to effectively estimate weak geometric distortions. For instance, the NIQSV+ metric can roughly distinguish three kinds of distortions, hole, stretching, and blurry distortion. Correspondingly, the scatter points present three clusters with different objective scores. However, due to the insufficiency of mixed weak distortions estimation, the corresponding objective scores of scatter points are close in each cluster, as shown in the subfigure of Figure 7. Hence, the objective scores calculated by the MLFA method can achieve higher consistency with human subjective perception.

Table 3 shows PLCC, SRCC, and RMSE of MLFA with state-of-the-art IQA metrics on the IETR DIBR image database, where the best results are highlighted in boldface. One can see that the performance of some representative metrics, such as NIQSV+ and CLGM, is poor. These metrics mainly measure limited and salient distortion types via decentralized attention. Thus, the defects, i.e., poor robustness for update 3D synthesized scenes, are easily exposed on the database without old-fashioned distortions. Among these metrics, the performance of SC-IQA metric is the best. However, its PLCC, SRCC, and RMSE are only 0.6856, 0.6423, and 0.1805. Comparatively, the MLFA method obtains the best performance on this database, i.e., PLCC, SRCC, and RMSE are 0.7378, 0.7036, and 0.1899. It validates that the MLFA method is effective and robust for various distorted scenes, especially including weak geometric distortions.

### 4.4. Generalization Ability

As a train-test-based quality model, the generalization ability is a persuasive robustness criterion. Therefore, we verify the generalization ability of our visual-entropy-guided MLFA method through a cross-experiment, where the best results are also marked in bold. Specifically, (1) the IETR DIBR image database is used when training the model, and the IRCCyN_IVC_DIBR_images database is used to test. (2) The IRCCyN_IVC_DIBR_images database is adopted to train and the IETR DIBR image database is used to test. Table 4 shows the performance comparison of our MLFA method and the other NR state-of-the-art synthesized IQA metrics. One can see that the proposed MLFA method acquires the best performance among these metrics. In addition, the performance of training on the IETR DIBR image database and testing on the IRCCyN_IVC_DIBR_images database is better than training on the IRCCyN_IVC_DIBR_images database and testing on the IETR DIBR image database. This is because the distortions of IRCCyN_IVC_DIBR_images database are old-fashioned, while the distortions of IETR DIBR image database are upgraded and more meticulous.

### 4.5. Impact of Training Percentages

To research how the amount of training data affects the performance of MLFA method, we execute the experiment via adopting different proportions of two DIBR image databases with 10% steps to train the model. Mainly, the image percentages of database used to train the model are set to five levels, i.e., 90%, 80%, 70%, 60% and 50%. All of the training–testing processes are operated 1000 times to get the median value, and the results are shown in Table 5. With the cut back of training data, the performance of the model gradually decreases. However, even with the lowest 50% training in the IRCCyN_IVC_DIBR_images database, we still get relatively good performance compared to most state-of-the-art synthesized IQA metrics, i.e., PLCC reaches 0.83. Moreover, on IETR DIBR image database, with only 50% training, our method outperforms the state-of-the-art metrics. These experiment results verify that our proposed MLFA method can still achieve better performance even if it uses less data for training.

### 4.6. Performance Analysis of a Multi-Layer Strategy

To illustrate the superiority of the visual-entropy-guided multi-layer strategy proposed in our method, we conduct a comparative experiment S1 with a single-layer strategy. In S1, the key region of the test image is firstly extracted. Then, the features of hole, deforming, blurry, and stretching are measured at the same level. Here, the MLFA method based on multi-layer strategy is denoted as S2.

Table 6 lists PLCC, SRCC, and RMSE of S1 and S2 on IRCCyN_IVC_DIBR_images and IETR DIBR image databases. Both S1 and S2 show good performance, which validate the integral effectiveness of feature extraction algorithms in our method. However, the performance of S1 is not poor but worse than S2, e.g., the PLCC value of S2 is about 0.02 higher than S1 on two databases. It suggests that putting the distortions of different visual stimuli at the same level will affect the accuracy of feature extraction to a certain extent. Therefore, the multi-layer strategy is conducive to further improving the performance of 3D synthesized IQA metrics.

### 4.7. Performance Analysis of Key Region Extraction

Table 5 shows the PLCC performance of *f_h_*, *f_def_*, *f_blu_*, *f_str_*, and *f_M_* with or without key region extraction. Specifically, we perform a comparative experiment denoted by S3. In S3, the process of key region extraction is canceled. Meanwhile, the scheme with key region extraction (i.e., MLFA method) is named S4. From Table 7, the PLCC results of various features in S3 and S4 on different databases can reflect the following two conclusions:

(1) S3 and S4 have similar PLCC performance on the bottom-up layer (i.e., *f_h_*). However, the performance of S3 is reduced on the top-down layer, especially for *f_M_*. Theoretically, most regions of the 3D synthesized images are not geometrically distorted. In S3, the features are extracted throughout the entire image, and the local geometric distortions are too subtle to be extracted. However, S4 adopts key region extraction, which highlights the regions of weak geometric distortion. Hence, the interference of most non-geometric distortion regions is effectively eliminated. The experimental data indeed verifies this theoretical explanation, i.e., the PLCC of S4 is nearly twice as high as S3 in *f_M_* on two databases.
(2) Different from *f_M_*, the PLCC performance of *f_def_*, *f_blu_*, and *f_str_* on the top-down layer is slightly affected by key region extraction. *f_M_* is a multi-dimensional feature and is obtained by concentrated attention. By contrast, *f_def_*, *f_blu_*, and *f_str_*, are single-dimensional features, and extracted from corresponding distortions via decentralized attention. Thus, the latter features are more distortion-specific, and insensitive to the regional interference in different scenes. The analysis is validated by the experimental results, which the PLCC of S3 slightly decreases within 0.04 compared to S4 in terms of *f_def_*, *f_blu_*, and *f_str_*.

In short, the experimental results on both two databases verify the effectiveness of the key region extraction on the top-down layer. In particular, the strategy of key region extraction plays a decisive role in the performance of *f_M_*, which means that the key region extraction is close relative to concentrated attention, and is a potential reason for the superiority of the overall model.

### 4.8. Feature Ablation Experiments

To analyze the contribution of the feature component, we also perform feature ablation experiments on the IRCCyN_IVC_DIBR_images database in which *f_h_*, *f_def_*, *f_blu_*, *f_str_*, and *f_M_* are permuted and combined into 17 models. The experimental results are listed in Table 8, and the best results are marked in bold. From extensive experimental results, two arguments can be made.

(1) M1–M5, composed of one feature, have poor performance, i.e., PLCC is below 0.7 roughly. In M6–M11, the feature components reach three, and the PLCC ranges from 0.7465 to 0.8174. For M12–M16, the feature components are increased to four, and the PLCC is further improved and stabilized in 0.8294 to 0.8538. In M17, PLCC is the best and equals 0.8757, when the feature components are five. The experimental data show that the performance increases in steps and gradually stabilizes with the addition of feature components. Hence, each feature is an essential part of the MLFA method and can significantly increase the accuracy and stability of the IQA model.
(2) Among these models, M12 and M17 are emphatically compared. In M12, the features are merely obtained by decentralized attention (as traditional distortion-classification-based 3D synthesized IQA metrics do). In M17, the features are acquired via feature integration theory, i.e., the interaction of decentralized attention and concentrated attention (as the MLFA method does). Obviously, the performance of M17 is better than M12, i.e., PLCC and SRCC are 0.0353 and 0.0988 higher than M12, and RMSE is 0.0145 lower than M12. The performance comparison demonstrates that the MLFA method, which uses the strategy of feature integration theory, achieves higher feature utilization and improves the consistency with the subjective scores.

**Figure 8:**
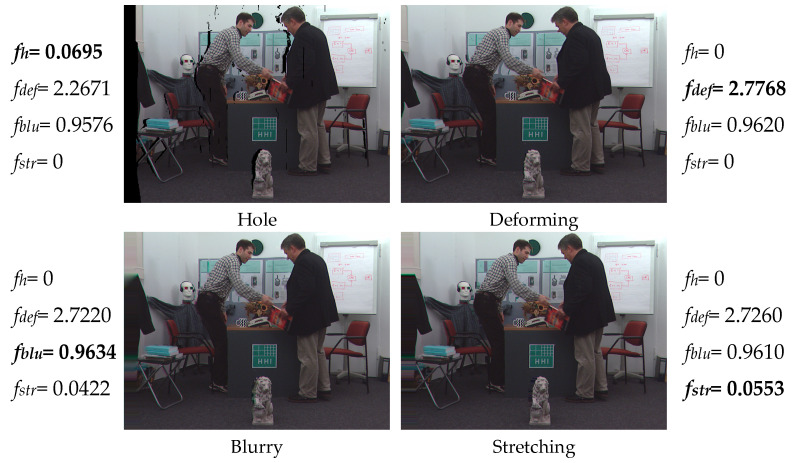
One-dimensional feature extraction of four images with different geometric distortions.

Figure 8 shows four images in the IRCCyN_IVC_DIBR_images database, which includes different geometric distortions. We extracted their features of the single-dimensional channel (i.e., *f_h_*, *f_def_*, *f_blu_*, *f_str_*) separately and listed the results. It can be seen that the proposed feature extraction method acquires the largest value in their corresponding images, as shown in bold. Moreover, if there are several kinds of distortions in an image, the MLFA model can still work very well. For example, the ‘blurry’ images also include some stretching distortion. The value of stretching feature is extracted as 0.0422 but less than 0.0553 of the ‘stretching’ image. This further verifies that the relationship between the proposed feature extraction model and their corresponding distortion is highly consistent, and the feature extraction model can reflect image distortion levels pretty well.

## 5. Conclusions

In this paper, we have proposed an NR IQA metric based on visual-entropy-guided MLFA for 3D synthesized images. Taking into account the stimulation of energy entropy to the human visual attention mechanism, different geometric distortions are divided into bottom-up layer and top-down layer. The ratio of 0-value pixels and the transition threshold are combined to calculate the hole feature on the bottom-up layer. In the meantime, based on key distorted region extraction, we adopt the interaction of decentralized and concentrated attentions to obtain the features of insignificant geometric distortions on the top-town layer. The final objective scores are obtained by regressing the features on multiple visual attention layers through RF. Extensive experiments have demonstrated that, compared with classical and state-of-the-art metrics, our MLFA method achieves better performance both on two public synthesized image databases and has a higher consistency with human subjective perception.

## Figures and Tables

**Table 1:** Influence of *n* on the performance of hole and non-hole distinction.

*n*	3	5	7	9	11
Standard deviation of hole	17.87	17.16	18.32	18.33	18.55
Standard deviation of non-hole	1.67	2.06	4.56	7.63	11.74
*T*	32	33	42	47	-
Computational time (s)	2.83	3.71	4.40	4.74	4.92

**Table 2:** Performance comparison of the proposed method with state-of-the-art metrics on the IRCCyN_IVC_DIBR images database.

Category	Distortion Type	Metric	PLCC	SRCC	RMSE
FR	2D traditional distortion	PSNR	0.4515	0.4589	0.5527
		SSIM [11]	0.4850	0.4368	0.5823
FR	3D synthesized distortion	VSQA [16]	0.5742	0.5233	0.5451
		3DSwIM [17]	0.6584	0.6156	0.5011
		ST-SIAQ [18]	0.6914	0.6746	0.4812
		EM-IQA [19]	0.7430	0.6282	0.4455
		MW-PSNR [20]	0.5622	0.5757	0.5506
		MP-PSNR [21]	0.6174	0.6227	0.5238
		SC-IQA [22]	0.8496	0.7640	**0.3511**
		IDEA [23]	0.7796	0.6652	0.3533
NR	3D synthesized distortion	APT [24]	0.7307	0.7157	0.4546
		MNSS [25]	0.7700	0.7850	0.4120
		OUT [26]	0.7243	0.7010	0.4591
		NR-MWT [28]	0.7343	0.5169	0.4520
		SET [30]	0.8586	0.8109	0.3015
		NIQSV [31]	0.6346	0.6167	0.5146
		NIQSV+ [32]	0.7114	0.6668	0.4679
		CLGM [33]	0.6750	0.6528	0.4620
		GANs-NRM [34]	0.8262	0.8072	0.3861
		Wang [35]	0.8112	0.7520	0.3820
		MLFA	**0.8757**	**0.8579**	0.4106

**Table 3:** Performance comparison of the proposed method with state-of-the-art metrics on the IETR DIBR image database.

Category	Distortion Type	Metric	PLCC	SRCC	RMSE
FR	2D traditional distortion	PSNR	0.6012	0.5356	0.1985
		SSIM [11]	0.4016	0.2395	0.2275
FR	3D synthesized distortion	VSQA [16]	0.5576	0.4719	0.2062
		ST-SIAQ [18]	0.3345	0.4232	0.2336
		EM-IQA [19]	0.5627	0.5670	0.2020
		MW-PSNR [20]	0.5301	0.4845	0.2106
		MP-PSNR [21]	0.5753	0.5507	0.2032
		SC-IQA [22]	0.6856	0.6423	**0.1805**
NR	3D synthesized distortion	APT [24]	0.4225	0.4187	0.2252
		MNSS [25]	0.3387	0.2281	0.2333
		OUT [26]	0.2007	0.1924	0.2429
		NR-MWT [27]	0.4769	0.4567	0.2179
		NIQSV [31]	0.1759	0.1473	0.2446
		NIQSV+ [32]	0.2095	0.2190	0.2429
		CLGM [33]	0.1146	0.0860	0.2463
		MLFA	**0.7378**	**0.7036**	0.1899

**Table 4:** Cross-validation of the proposed MLFA method and the NR state-of-the-art metrics on IETR DIBR image database and IRCCyN_IVC_DIBR_images database.

Training Database	Testing Database	Method	PLCC	SRCC	RMSE
IETR DIBR image	IRCCyN_IVC_DIBR_images	APT	0.6745	0.5817	0.4916
		MNSS	0.6539	0.6147	0.5037
		NIQSV	0.4989	0.0889	0.5494
		NIQSV+	0.5921	0.2680	0.5365
		MLFA	**0.8645**	**0.8562**	**0.3945**
IRCCyN_IVC_DIBR_images	IETR DIBR image	APT	0.3838	0.2198	0.2249
		MNSS	0.2829	0.2196	0.2335
		NIQSV	0.1216	0.0839	0.2416
		NIQSV+	0.0292	0.0569	0.2433
		MLFA	**0.7046**	**0.6720**	**0.2181**

**Table 5:** Performances of the proposed MLFA method with different training percentages.

Database	Training–Testing	PLCC	SRCC	RMSE
IRCCyN_IVC_DIBR_images	90–10%	0.8895	0.8585	0.2967
	80–20%	0.8757	0.8579	0.3106
	70–30%	0.8620	0.8330	0.3871
	60–40%	0.8467	0.8073	0.4339
	50–50%	0.8303	0.7970	0.5010
IETR DIBR image	90–10%	0.7473	0.7158	0.1642
	80–20%	0.7378	0.7036	0.1899
	70–30%	0.7180	0.6845	0.1928
	60–40%	0.7055	0.6644	0.2027
	50–50%	0.6899	0.6473	0.2092

**Table 6:** Performance comparison of S1 and S2 on two databases.

Scheme	IRCCyN_IVC_DIBR_Images			IETR DIBR Image		
	PLCC	SRCC	RMSE	PLCC	SRCC	RMSE
S1	0.8558	0.8004	0.4269	0.7133	0.6861	0.2061
S2	0.8757	0.8579	0.4106	0.7378	0.7036	0.1899

**Table 7:** PLCC comparison with or without key region extraction.

Database	Scheme	*f_h_*	*f_def_*	*f_blu_*	*f_str_*	*f_M_*
IRCCyN_IVC_DIBR_images	S3	0.5409	0.5760	0.6248	0.3956	0.3535
	S4	0.5416	0.6108	0.6358	0.4331	0.6906
IETR DIBR image	S3	0.4278	0.2972	0.4092	0.3367	0.2094
	S4	0.4271	0.3365	0.4165	0.3681	0.4544

**Table 8:** Performance of different feature components on the IRCCyN_IVC_DIBR images database.

Models	Features					IRCCyN_IVC_DIBR_Images		
	*f_h_*	*f_def_*	*f_blu_*	*f_str_*	*f_M_*	PLCC	SRCC	RMSE
M1	√					0.5416	0.3670	0.7050
M2		√				0.6108	0.3543	0.6248
M3			√			0.6358	0.5375	0.6420
M4				√		0.4331	0.3829	0.7605
M5					√	0.6906	0.5639	0.6022
M6	√	√	√			0.8174	0.7558	0.4518
M7	√	√		√		0.7465	0.6680	0.5366
M8	√	√			√	0.8029	0.7301	0.4856
M9	√		√	√		0.8103	0.7085	0.4698
M10	√		√		√	0.7895	0.7088	0.4850
M11	√			√	√	0.7781	0.6887	0.5049
M12	√	√	√	√		0.8404	0.7591	0.4251
M13	√	√	√		√	0.8378	0.7735	0.4353
M14	√	√		√	√	0.8294	0.7511	0.4537
M15	√		√	√	√	0.8373	0.7598	0.4497
M16		√	√	√	√	0.8538	0.7997	0.4146
M17	√	√	√	√	√	**0.8757**	**0.8579**	**0.4106**
